# Towards broad-spectrum protection: the development and challenges of combined respiratory virus vaccines

**DOI:** 10.3389/fcimb.2024.1412478

**Published:** 2024-06-05

**Authors:** Yang Wang, Xiaotong Wei, Yang Liu, Shengfeng Li, Weiqi Pan, Jun Dai, Zifeng Yang

**Affiliations:** ^1^State Key Laboratory of Respiratory Disease, National Clinical Research Center for Respiratory Disease, Guangzhou Institute of Respiratory Health, The First Affiliated Hospital of Guangzhou Medical University, Guangzhou, China; ^2^Guangzhou National Laboratory, Guangzhou, China; ^3^Guangzhou Customs Technology Center, Guangzhou, China; ^4^Respiratory Disease AI Laboratory on Epidemic and Medical Big Data Instrument Applications, Faculty of Innovation Engineering, Macau University of Science and Technology, Macao, Macao SAR, China

**Keywords:** combined vaccine, SARS-CoV-2, COVID-19, influenza virus, RSV, mRNA vaccine, subunit vaccine, viral vector vaccine

## Abstract

In the post-COVID-19 era, the co-circulation of respiratory viruses, including influenza, SARS-CoV-2, and respiratory syncytial virus (RSV), continues to have significant health impacts and presents ongoing public health challenges. Vaccination remains the most effective measure for preventing viral infections. To address the concurrent circulation of these respiratory viruses, extensive efforts have been dedicated to the development of combined vaccines. These vaccines utilize a range of platforms, including mRNA-based vaccines, viral vector vaccines, and subunit vaccines, providing opportunities in addressing multiple pathogens at once. This review delves into the major advancements in the field of combined vaccine research, underscoring the strategic use of various platforms to tackle the simultaneous circulation of respiratory viruses effectively.

## 1 Introduction

The global community is currently confronting a severe public health crisis due to the co-circulation of respiratory viruses, notably the influenza virus, the respiratory syncytial virus (RSV), and SARS-CoV-2 virus. Seasonal influenza alone impacts 5%~10% of adults and 20%~30% of children worldwide annually, resulting in 3~5 million severe cases and approximately 290,000 to 650,000 deaths (Houser and Subbarao, 2015; Iuliano et al., 2018). RSV infection is a common cause of lower respiratory tract illness among infants, young children and older adults (Organization WH, 2019). It leads to an estimated 33 million cases and over 3 million hospitalizations and about 59,600 deaths in children under 5-year annually (Li et al., 2022). In the United States, among adults aged 65 and older, it is estimated that there are 160,000 hospitalizations and 13,000 deaths annually associated with RSV (McLaughlin et al., 2022a). The emergence of coronavirus disease 2019 (COVID-19) pandemic, caused by SARS-CoV-2, has further complicated the landscape, with at least 775 million cases and approximately 7 million deaths reported globally to date (Organization WH, 2024). While the non-pharmaceutical interventions for the COVID-19 pandemic initially led to a decrease in the spread of respiratory diseases, the relief of these measures has prompted a rebound in respiratory infections. In the 2022 winter season, the simultaneous circulation of influenza, RSV, and SARS-CoV-2 viruses (a phenomenon referred to as “tripledemics”) severely strained healthcare resources (Subspecialty Group of Respiratory Diseases tSoPCMA et al., 2023). In 2023, an early onset of the flu season combined with heightened RSV activity and persistent COVID-19 cases exerted significant strain on China’s healthcare system, especially affecting pediatric services (Organization WH, 2023). Moreover, co-infection with these viruses, such as influenza and SARS-CoV-2, has been well-documented, with reported prevalence rates ranging from 0.2% to 48% (Kim et al., 2020; Dadashi et al., 2021; Stowe et al., 2021; Tang et al., 2022). Such co-infections typically lead to more severe and prolonged illnesses (Liang et al., 2024; Adams et al., 2022; Choudhary et al., 2022; Yan et al., 2023).

**Figure 1:**
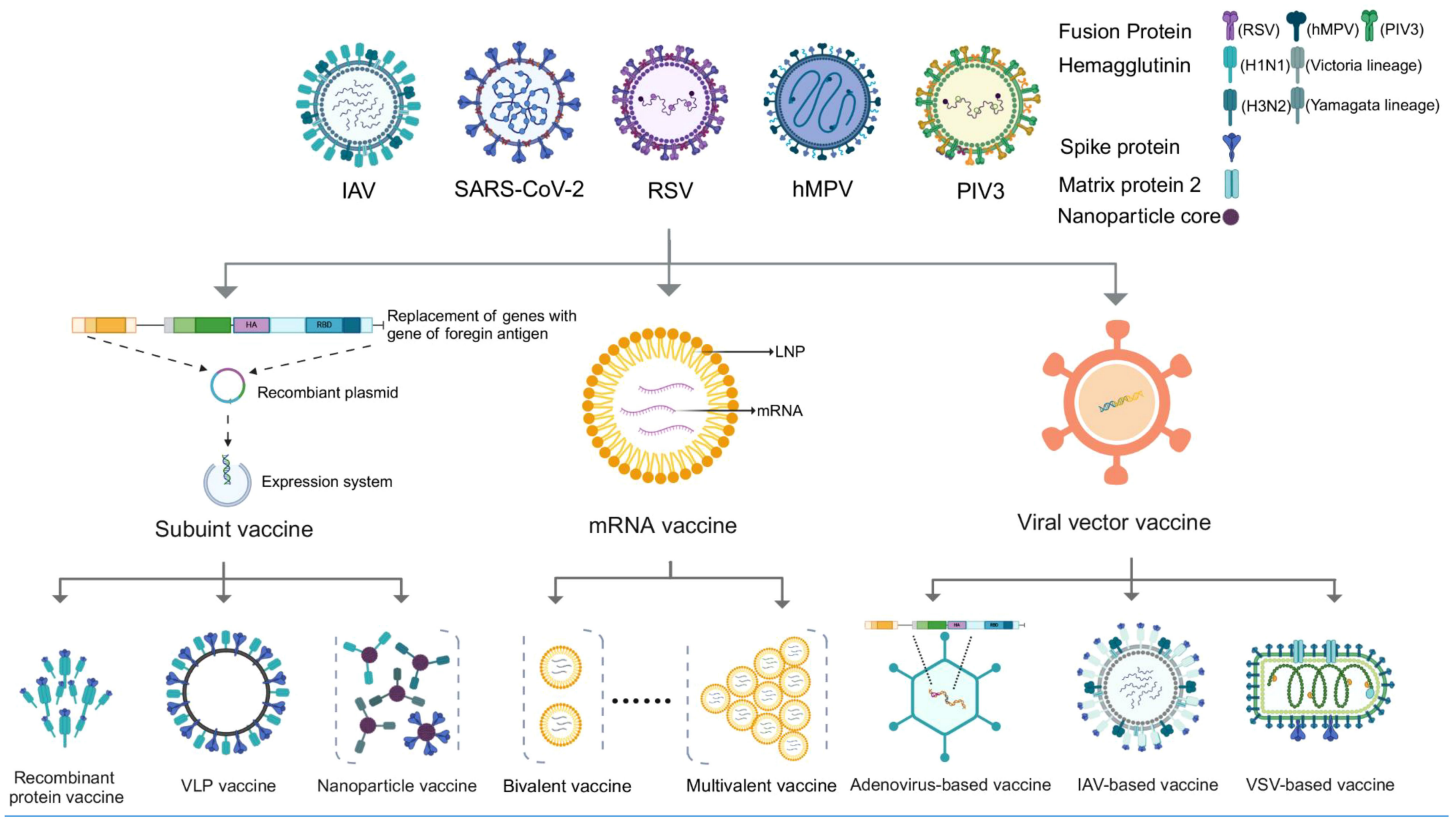
Various vaccine platforms considered for combined vaccines against respiratory viruses.

Vaccines play a crucial role in combating infectious diseases, yet respiratory viruses such as influenza, SARS-CoV-2, and RSV pose unique challenges. These viruses require frequent, sometimes annual, vaccinations due to quickly fading vaccine-induced immunity and/or their rapid mutation rates, which allows them to escape neutralizing antibodies (Wang et al., 2022; Morens et al., 2023). This scenario could necessitate multiple vaccinations each year. In response, combination vaccines offer a strategic advantage by merging multiple antigens into a single dose, enabling the simultaneous prevention of various diseases. Additionally, combination vaccines offer several social benefits: 1) reducing the need for multiple injections and minimizing side effects; 2) decreasing healthcare visits for both recipients and guardians; 3) simplifying vaccine management and storage, and reducing the risk of needlestick injuries for healthcare providers; 4) improving the timeliness and coverage of vaccination (Maman et al., 2015; Lennon et al., 2022). Reflecting these advantages, a recent large-scale survey in the United States (n = 12,887) found that 50% of respondents were in favor of a combined COVID-19/influenza vaccine (Lennon et al., 2022). Therefore, in this review, we summarize the major advances in the field of combined vaccines for respiratory viruses, including influenza, SARS-CoV-2, RSV, and others (**Figure 1**, **Table 1**).

**Table 1:** Summary of the Progress of Combined Vaccines Against Respiratory Viruses.

Platform	Target	Name	Antigen	Status	Reference
mRNA vaccines	Influenza and SARS-CoV-2	AR-CoV/IAV	Full-length HA, RBD	Preclinical development	(Ye et al., 2022)
		mRNA-1073	Full-length HA, Spike protein	Phase I, II	NCT05375838
		mRNA-1083	Full-length HA, RBD and NTD	Phase I, II, III	NCT06097273
		qIRV (22/23)/bivalent BNT162b2	Full-length HA, Spike protein	Phase I, II	NCT06696734
		FLUCOV-10	Full-length HA, Spike protein	Preclinical development	(Wang et al., 2020)
	Influenza and RSV	mRNA-1045	Full-length HA, pre-F protein	Phase I	NCT05585632
	SARS-CoV-2 and RSV	SF-LNP/S-LNP+F-LNP	Spike protein, F protein	Preclinical development	(Wu et al., 2024)
	Influenza, RSV and SARS-CoV-2	mRNA-1230	Full-length HA, pre-F protein, Spike protein	Phase I	(Moderna, 2023b)
	hMPV and PIV3	mRNA-1653	F proteins	Phase I	(August et al., 2022)
Recombinant protein vaccines	Influenza and SARS-CoV-2	FLU-COVID HA+ Omicron S HA1 trimer+ RBD trimer H1Delta	Full-length HA, Spike protein Full-length HA, Spike protein HA1 trimer, RBD trimer HA stalk, RBD	Preclinical development Preclinical development Preclinical development Preclinical development	(Huang et al., 2024) (Zhang et al., 2024) (Shi et al., 2022) (Li et al., 2023)
Virus-like-particle vaccine	Influenza and SARS-CoV-2	VLP-RBD-GM-CSF-(IL-12)	Inactivated IAV, RBD	Preclinical development	(Bommireddy et al., 2022)
Nanoparticle vaccine	Influenza and SARS-CoV-2	qNIV/CoV2373	Full-length HA, Spike protein	Phase I, II	(Massare et al., 2021) (Novavax, 2022)
Influenza-based vaccines	Influenza and SARS-CoV-2	TM-RBD-HA Pneucolin (dNS1-RBD) Flu-RBD ΔNA(RBD)-Flu	Live attenuated IAV, RBD Live attenuated IAV, RBD Inactivated IAV, RBD Attenuated IAV, RBD	Preclinical development Phase I, II, III (Emergence use only) Preclinical development Preclinical development	(Chaparian et al., 2022) (Chen et al., 2022; Zhu et al., 2022; Zhang et al., 2023; Zhu et al., 2023) (Wang et al., 2023) (Loes et al., 2020)
	Influenza and RSV	NS-F/NS-2AF/NS-2AsF/sF-NS	Attenuated IAV, F protein	Preclinical development	(Pulkina et al., 2023)
VSV-based vaccines	Influenza and SARS-CoV-2	V-EM2/ERBD	M2e, RBD	Preclinical development	(Ao et al., 2022)
Adenoviral vectors vaccines	Influenza and SARS-CoV-2	Adc68-CoV/Flu	HA stalk, RBD	Preclinical development	(Cao et al., 2022)

M2e: The Matrix 2 ectodomain of influenza virus;

NTD: The N-Terminal domain of the SARS-CoV-2 spike protein;

HA1 trimer: trimerized hemagglutinin 1 of influenza virus;

RBD trimer: trimerized receptor binding site of SARS-CoV-2 spike protein;

IAV: influenza A virus.

## 2 Combined mRNA-based vaccines

Before the COVID-19 pandemic, gene-based vaccines had not received authorization for public use. This changed dramatically during the pandemic with the rapid development and widespread deployment of COVID-19 mRNA vaccines. Notably, the Pfizer-BioNTech BNT162b2 vaccine and Moderna mRNA-1273, both administered in two doses, demonstrated efficacies of 95% (n=18,160) and 94.1% (n=12,273) in preventing COVID-19, respectively (Polack et al., 2020) (Baden et al., 2021). These vaccines have been extensively validated for safety and efficacy in preventing serious illness and fatalities (Rzymski et al., 2023). Additionally, the mRNA vaccine platform stands out for its flexibility to encode various antigens and its ease in combining them into a single formulation. Recently, a universal influenza mRNA vaccine was developed, integrating different mRNA components to encode all 20 influenza subtypes (Arevalo et al., 2022). This capability is particularly beneficial for combating co-circulation of multiple respiratory viruses within a single dose (Whitaker et al., 2023). The production attributes of mRNA vaccines—namely their rapid development, scalability, and cost-effectiveness—further enhance their appeal (Whitaker et al., 2023). Currently, the research and development of combined mRNA vaccines targeting various respiratory viruses are advancing rapidly, progressing from pre-clinical studies to clinical trials at an impressive pace (Pardi et al., 2018; Gote et al., 2023).

### 2.1 Combined mRNA vaccines against influenza and SARS-CoV-2

During the initial wave of the COVID-19 pandemic, there was a notable occurrence of co-infections with influenza (Maltezou et al., 2023), catalyzing the early development of bivalent mRNA vaccines targeting both viruses. Ye et al. reported a combined mRNA-based vaccine named AR-CoV/IAV, which includes mRNAs encoding the hemagglutinin (HA) protein of the seasonal influenza A/H1N1 virus and the receptor-binding domain (RBD) of SARS-CoV-2 virus, each encapsulated in lipid nanoparticles (LNPs) separately (Ye et al., 2022). Their findings indicated that in a mouse model, the vaccine efficiently elicited the production of IgG and neutralizing antibodies against both antigens, alongside CD4+ and CD8+ T cell responses. Furthermore, it provided complete protection against both influenza and SARS-CoV-2, including co-infection scenarios with these viruses following two doses of vaccination (Ye et al., 2022). Several manufacturers are making efforts for combined mRNA vaccines, including Moderna, which has two such mRNA vaccines in clinical trials targeting both SARS-CoV-2 and influenza. The mRNA-1073 vaccine, a 5-valent mRNA vaccine encoding the HAs of four seasonal influenza A and B viruses along with the full-length spike protein of one SARS-CoV-2 virus, is currently in Phase 1/2 trials with participants aged 18-75 (NCT05375838). Concurrently, the mRNA-1083 vaccine, which includes mRNA components for four seasonal influenza viruses and mRNA components encoding the RBD and N-terminal domain of SARS-CoV-2 spike protein, is being evaluated in two age-specific cohorts: cohort A with healthy adults over 65 years, and cohort B with healthy adults between 50 to 65 years (NCT06097273). Also, BioNTech is similarly working on a combined vaccine for SARS-CoV-2 and influenza, now in a Phase II clinical trial (NCT05596734).

Considering the high fatality rate of zoonotic influenza virus infections, along with the concerns for their potential pandemic risks (Wang et al., 2017; Dong J. et al., 2020; Wang et al., 2020; Guan et al., 2023; Wang et al., 2024b). More recently, Wang et al. have developed a 10-valent mRNA vaccine candidate called FLUCOV-10 (Wang et al., 2024a). This vaccine encodes the full-length HAs of four seasonal influenza viruses and two avian influenza viruses, in addition to the full-length spike proteins of the ancestral SARS-CoV-2 and its three Omicron subvariants (Wang et al., 2024a). After administering two doses to mice, the FLUCOV-10 vaccine was found to effectively induce neutralizing antibodies and Th1-biased cellular immunity against each vaccine component, offering broad protection against lethal challenges from both matched and heterologous strains of influenza and SARS-CoV-2 (Wang et al., 2024a).

### 2.2 Other efforts of combined mRNA vaccines against respiratory viruses

The 2022’s tripledemic and the authorization of RSV vaccine based on the pre-fusion (pre-F) conformation of the RSV fusion (F) protein have prompted the development of combined respiratory virus vaccines to include RSV (McLellan et al., 2013). The mRNA vaccine platform offers promising opportunities for RSV vaccines. For example, Moderna’s mRNA-1777, which encodes RSV pre-F protein, has shown to be well-tolerated and enhance RSV F-specific humoral and cellular immunity both in healthy younger and older adults. Similarly, Moderna’s mRNA-1345, has demonstrated 83.7% efficacy against RSV associated lower respiratory tract disease with just a single dose. These vaccines have exhibited favorable safety profiles and immunogenicity in clinical trials (Aliprantis et al., 2021; Qiu et al., 2022; Moderna, 2023a; Shaw et al., 2024). Furthermore, Moderna has developed two combination vaccines that target RSV and other respiratory viruses: mRNA-1045, aimed at providing protection against seasonal influenza and RSV; and mRNA-1230, designed to protect against seasonal influenza, SARS-CoV-2, and RSV. A phase 1 study of these vaccines, being tested in healthy adults aged 50-75 years, is currently in progress (NCT05585632) (Moderna, 2023b). Wu et al. also developed a combined mRNA vaccine targeting both SARS-CoV-2 and RSV. They evaluated the effectiveness of delivering spike mRNA and pre-F mRNA in separate LNPs versus in a single LNP, and they found that there was no significant difference in protein expression and IgG antibody induction between these approaches (Wu et al., 2024).

Human metapneumovirus (hMPV) is a noteworthy virus associated with acute respiratory illness (ARI) and infectious of the upper and lower respiratory tract across all ages (Howard et al., 2018). Parainfluenza virus type 3 (PIV3) is a common factor in respiratory illness among infants and children (Boonyaratanakornkit et al., 2021). Currently, there are no approved vaccines or antiviral strategies for preventing hMPV and PIV3. A bivalent vaccine named mRNA-1653 is an investigational combination of mRNA-based vaccines that encode both the full-length membrane-anchored fusion proteins of hMPV and PIV3 (August et al., 2022). The first-in-human study showed that this vaccine was safe, well-tolerated, and highly immunogenic.

## 3 Combined subunit vaccines

Subunit vaccines incorporate only selected antigens—key components (e.g., viral surface proteins) of pathogens identified for their ability to strongly elicit humoral and cellular immune responses (Bill, 2015). This targeted approach minimizes the risk of adverse effects commonly associated with whole-pathogen vaccines and streamlines the manufacturing process (Roper and Rehm, 2009; Bayani et al., 2023). However, because these isolated antigens may not be potent enough to induce robust and long-lasting immunity, adjuvants are often added to enhance the immune response (Zhao et al., 2023). The efficacy and adaptability of subunit vaccines are underscored by their successful application in diverse pathogens, including influenza (Vogel and Manicassamy, 2020), SARS-CoV-2 (Dong Y. et al., 2020; Heidary et al., 2022), and RSV (Papi et al., 2023). Utilizing various platforms, such as recombinant protein vaccines (Liu et al., 2021; Chavda et al., 2024), virus-like particles (VLPs) (Sun et al., 2022), and nanoparticles (Pilkington et al., 2021), these vaccines have demonstrated considerable promise, with several vaccines receiving approval for human use or currently undergoing clinical trials. Moreover, subunit vaccines serve as a highly adaptable platform for the development of combined vaccines, capable of targeting multiple strains of viruses like human papillomavirus (HPV) and influenza (Liu et al., 2021; Williamson, 2023). This versatility has prompted considerable research efforts dedicated to exploring combined vaccines against multiple respiratory viruses.

### 3.1 Recombinant protein vaccine

Approved recombinant protein vaccines for influenza, SARS-CoV-2, and RSV utilized their unique trimeric class I transmembrane glycoproteins—HA, spike, and pre-F proteins, respectively (Sanders and Moore, 2021; Che et al., 2023). One approach to develop combined protein vaccines is by mixing functional recombinant proteins together. Huang et al. developed a trivalent vaccine for flu and COVID-19 by integrating full-length, computationally optimized broadly reactive antigens (COBRA) from H1N1 and H3N2 influenza viruses along with the SARS-CoV-2 spike protein, combined with the AddaVax adjuvant (Huang et al., 2024). Each protein was produced in HEK293F suspension cells, with its transmembrane domain truncated and replaced by a T4 foldon domain, an Avitag, and a 6x His-tag for the purpose of purification. They revealed that this combined vaccine elicited virus-specific antibodies, HI antibodies, and neutralizing antibodies at levels comparable to those from mono vaccines, effectively protecting K18-hACE2 transgenic mice against infections from both H1N1 influenza and SARS-CoV-2 (Huang et al., 2024). Zhang et al. created a bivalent recombinant protein vaccine targeting both influenza H1N1 and the SARS-CoV-2 Omicron variant, which was adjuvanted with MF59 (Zhang et al., 2024). Their findings showed that this vaccine induced strong responses of IgG, IgG1, and IgG2a antibodies, in addition to HI antibodies against influenza H1N1 and neutralizing antibodies against SARS-CoV-2 Omicron (Zhang et al., 2024). Instead of using full-length proteins, Shi et al. focused on the trimerized RBD of SARS-CoV-2 and the HA head (HA1) of influenza H1N1 to craft a bivalent vaccine (Shi et al., 2022). This strategy targeted the most immunogenic and receptor-binding portions of the proteins, resulting in a vaccine that demonstrated strong immunogenicity in mouse models, with high neutralizing antibody levels and a balanced Th1/Th2 cellular immune response (Shi et al., 2022).

Another approach to creating combined recombinant protein vaccines involves the chimeric fusion of different immunogenic proteins. Li et al. engineered a novel strategy by fusing the RBD from the SARS-CoV-2 Delta variant to the stalk of a headless H1 hemagglutinin (HA), resulting in a trimerized chimeric soluble protein vaccine. This innovative vaccine was successful in eliciting high and long-lasting neutralizing antibody responses, providing effective and broad protection against both matched and heterologous challenges from influenza and SARS-CoV-2 viruses (Li et al., 2023).

### 3.2 Virus-like-particle vaccines

VLPs vaccine is based on the virus’s special characteristic that its structural proteins, envelope proteins, and capsid proteins can self-assemble along or collectively without a viral genome, based on this theory, any viruses can be exploited to develop VLPs (Ao et al., 2022). In addition to its ability of presenting native membrane-bound conformation, the technology of VLPs is cost-effective in production and advantages in targeting selective antigens, showing remarkable safety and immunogenicity (Roldao et al., 2010). Bommireddy et al. innovatively conjugated the SARS-CoV-2 RBD, whose N terminus attached with GM-CSF and C terminus with glycosylphosphatidylinositol (GPI), into influenza envelope, VLPs using GPI-anchor mediated protein transfer, with the option of including GPI -IL-12 (Bommireddy et al., 2022). The cytokines GM-CSF and IL-2 function as adjuvants, enhancing immunogenicity by recruiting and activating crucial immune cells (Dranoff, 2003; Metzger, 2009). The GPI-anchor facilitates the integration of exogenous proteins into the lipid bilayer of influenza VLPs or amphiphilic micro/nanoparticles, enabling the direct presentation of viral antigens to the immune system, inducing a swift and pronounced immune response (Bommireddy et al., 2022).

### 3.3 Nanoparticle vaccines

Novavax developed a combined nanoparticle vaccine for COVID-19 and seasonal influenza, named COVID-19–NanoFlu, utilizing the full-length, stabilized recombinant SARS-CoV-2 spike protein (NVX-CoV2373) and four full-length recombinant HA proteins from four seasonal influenza viruses (NanoFlu™) as antigens (Massare et al., 2021). The recombinant proteins were expressed using a baculovirus–insect cell system, the resulting nanoparticles were assembled around polysorbate 80 cores to form one to multiple trimerized protein rosettes, and the vaccine was adjuvanted with Matrix-M (Massare et al., 2021). Tested in ferrets and hamsters, the vaccine induced antibodies against both viruses at levels comparable to those offered with each vaccine individually and protected the animals from a SARS-CoV-2 challenge (Massare et al., 2021). A phase I/II trial, involving 642 healthy adults aged 50–70, was conducted across 12 Australian sites (NCT04961541) and concluded successfully, showing the vaccine to be well-tolerated and immunogenic (Novavax, 2022). Following this, a phase 2 trial began in January 2023, targeting 2300 adults aged 50–80 (NCT05519839).

## 4 Combined viral vector vaccines

The widespread adoption of adenoviral vector vaccines during the COVID-19 pandemic highlighted their efficacy, safety, and ability to elicit strong immune responses (Hanke, 2022), motivating the investigation into diverse viral vectors for fighting respiratory viruses, including adenoviral (Dranoff, 2003; Cao et al., 2022), influenza (Loes et al., 2020; Chaparian et al., 2022; Chen et al., 2022; Zhu et al., 2022; Pulkina et al., 2023; Wang et al., 2023; Zhang et al., 2023; Zhu et al., 2023), and VSV vectors (Ao et al., 2022; Liu et al., 2022). Viral vector vaccines are capable of not only eliciting cellular and humoral immune responses but also stimulating cytokine and chemokine production as a part of the proinflammatory response (Quinn et al., 2015). Furthermore, viral vector vaccines offer the convenience of non-invasive administration methods, such as nasal sprays and nebulization, coupled with the benefits of a low incidence of adverse reactions and reduced vaccine hesitancy. Additionally, the mucosal immune response that they elicit plays a crucial role in effectively preventing the transmission of respiratory viruses (Muster et al., 1995; Moreno-Fierros et al., 2020).

### 4.1 Adenoviral vector vaccines

During the COVID-19 pandemic, adenoviral vector vaccines were widely used, with billions of doses administered globally, affirming their safety, immunogenicity, and efficacy (McCann et al., 2022). Cao et al. developed a combined vaccine against flu and COVID-19, named AdC68-CoV/Flu (Cao et al., 2022), utilizing Chimpanzee adenovirus serotype-68 (AdC68) for its low pre-existing immunity and positive safety profile. This vaccine incorporates a fusion immunogen combining the SARS-CoV-2 RBD region with the influenza H7N9 HA stalk region and utilizes ferritin to enhance immunogenicity by forming nanoparticles (Jiang et al., 2020). The HA stalk is expected to produce cross-reactive antibodies because of its conserved nature across influenza viruses, and it also stabilizes the RBD for native trimer formation and attachment to ferritin. In mouse studies, the vaccine successfully triggered antibody responses against both influenza H7N9 and SARS-CoV-2, RBD-specific T cell responses, and offered complete protection against these viruses. However, the vaccine induced limited immune response and protection against H3N2 and heterologous SARS-CoV-2 variants (e.g., B.1.351, P.1, and B.1.627), highlighting the challenge in providing broad protection against seasonal influenza and antigenically diverse SARS-CoV-2 strains (Cao et al., 2022).

### 4.2 Influenza-based vaccines

The influenza-based vaccine platform, designed to fight flu and other respiratory viruses, works by integrating foreign proteins into attenuated or replication-deficient influenza viruses, thereby eliciting strong immune responses against both pathogens. Various teams have employed different strategies using the influenza virus as a vector to design combined vaccines against flu and COVID-19. Chaparian et al. engineered a chimeric virus by inserting fusion fragments of the SARS-CoV-2 RBD and the transmembrane domain of the influenza neuraminidase (NA) protein into the influenza HA gene (Chaparian et al., 2022). This design allows the influenza virus envelope to display influenza HA proteins and the SARS-CoV-2 RBD domain simultaneously. This chimeric virus, either in an inactivated form or as a live attenuated vaccine, successfully induces protective immune responses against both influenza and SARS-CoV-2 in mice (Chaparian et al., 2022). Similarly, Loes et al. substituted the NA gene coding region in the influenza genome with the membrane-anchored SARS-CoV-2 RBD region, creating a multicycle replicating influenza virus vector vaccine that is NA-deleted and targets both flu and COVID-19 (Loes et al., 2020).

Notably, Xia’s group has made significant strides in developing an influenza vector nasal spray vaccine, showing potential as a combined vaccine candidate against both influenza and SARS-CoV-2 (Chen et al., 2022; Zhang et al., 2023). They replaced the non-structural-1 (NS1) gene of influenza with the SARS-CoV-2 RBD region, creating a NS1-deleted, multicycle replication, live-attenuated influenza-based SARS-CoV-2 vaccine named dNS1-RBD (Pneucolin^®^). Preclinical studies in hamsters have shown that this nasal vaccine not only triggers systemic humoral and cellular immune responses but also stimulates local innate immunity, secretory IgA (sIgA), and tissue-resident memory T cells in the respiratory tract. It has proven effective in mitigating lung pathology and preserving body weight after vaccination when challenged either shortly or several months later (Chen et al., 2022; Zhang et al., 2023). Remarkably, it provides coverage against various SARS-CoV-2 variants, including the Omicron variant, and offers cross-protection against H1N1 and H5N1 influenza viruses (Chen et al., 2022; Zhang et al., 2023). Phase 1-2 clinical trials in adults have shown that the vaccine is safe and effective against the Omicron variant (Zhu et al., 2022). Moreover, in phase III clinical trials, the vaccine showed a protection rate of about 65% against symptomatic infections by the Omicron variant, surpassing the real-world effectiveness of intramuscular vaccines against this variant (Zhu et al., 2023).

Wang et al. adopted a conjugation method to create an influenza-based combination vaccine (Wang et al., 2023). They anchored the SARS-CoV-2 RBD onto the inactivated influenza virus surface using a bio-orthogonal click reaction between dibenzo cyclooctyne (conjugated to viral envelop) and azido (linked to the RBD). In hamster model, two doses of this vaccine induced robust humoral and cellular immune responses, including viral-specific neutralizing antibodies, and offered protection against both pathogens, evidenced by reduced viral replication in the BALF and diminished lung lesions in the vaccinated hamsters (Wang et al., 2023).

Pulkina et al. developed an RSV vaccine by using the influenza vector platform. They created four vaccines that inserted the RSV F gene into modified NS segments by diverse structures for either cytosolic accumulation or extracellular delivery of F proteins. They found that the vaccine with an IgGκ signal peptide for extracellular delivery markedly increased T-cell immunity and effectively protected against RSV with just one intranasal vaccination (Pulkina et al., 2023).

### 4.3 VSV-based vaccines

The recombined vesicular stomatitis virus (rVSV)-based vaccine platform, which replaces the VSV-glycoprotein (G) with foreign immunogens, enables a rapid and robust immune response from a single dose and provides protection against various pathogens, such as Ebola, Zika, HIV, and Nipah viruses (Rose et al., 2001; DeBuysscher et al., 2014; Marzi et al., 2015; Emanuel et al., 2018). Ao et al. developed three versions of attenuated rVSV-based vaccines targeting both SARS-CoV-2 and influenza virus (Ao et al., 2022). They engineered three chimeric proteins to simultaneously express specific regions of the SARS-CoV-2 spike protein and the highly conserved M2 ectodomain (M2e) of influenza. Among these bivalent vaccines, V-EM2e/SPΔC1, containing the full-length spike protein, and V-EM2e/SPΔC2, with the S2- deleted spike protein, were particularly effective. These vaccines induced strong humoral and cellular immune responses, offering significant protection in hamsters or mice against challenges with the SARS-CoV-2 Delta variant and H1N1 and H3N2 influenza infections, outperforming the V-EM2/ERBD vaccine that only targets the spike protein’s RBD (Ao et al., 2022).

## 5 Discussion

The objective of developing a combined mRNA vaccine is to achieve broad protection against multiple targets. A significant challenge in this endeavor is ensuring that each component of the vaccine is both immunogenic and effective. Evidence from licensed influenza vaccines and a quadrivalent seasonal influenza mRNA vaccine candidate indicates a tendency for lower responses to influenza B strains compared to the A strains (Cowling et al., 2020; Lee et al., 2023; Reneer et al., 2023). Similarly, in our FLUCOV-10 vaccine candidate, we observed varied immunogenicity levels among different influenza HA components, with the influenza B mRNA components exhibiting notably lower immunogenicity (Wang et al., 2024a). Further analysis revealed that this discrepancy is due to the inherently low immunogenicity of influenza B HA and its interactions with other vaccine components (Wang et al., 2024a). Therefore, future development of combined vaccines should pay special attention to adjusting the formulation to optimize the immunogenicity and efficacy of each component.

Among the various platforms used for combined vaccines, each displays unique advantages. For instance, the mRNA-based vaccine platform offers rapid manufacturing times and the ability to incorporate multiple pathogens or serotypes flexibly. A recent breakthrough involved validating a universal mRNA-based influenza vaccine that targets all 20 known influenza virus subtypes in animal models (Arevalo et al., 2022), making it a promising strategy for a pan-respiratory virus vaccine. Moreover, mRNA vaccines aimed at SARS-CoV-2 and quadrivalent seasonal influenza (Clinical trial No.: NCT05375838, NCT06097273, and NCT05596734), or even a combination including SARS-CoV-2, RSV, and quadrivalent seasonal influenza viruses (NCT05585632), have pioneered their way into clinical trials, marking a significant advancement in vaccine technology. As for the subunit vaccine platform, it is recognized for its cost-effective production and ability to present high-density, properly folded antigens that ensure safety and immunogenicity (Wang et al., 2016; Dong Y. et al., 2020). However, combining different antigens from various viruses into a single vaccine poses challenges due to chemical incompatibility and potential immunological interference. While some approaches have sought to address these issues by fusing different antigens into single recombinant protein, no fusion protein vaccine has yet received approval, to the best of our knowledge. This is partly because the fusion of different antigens can compromise the safety and tolerability of the resulting vaccine (Vartak and Sucheck, 2016). On the other hand, VLPs and nanoparticles have intrinsic properties of heterogeneity and highly ordered structural organization, which may ensure the stable structure of each antigen in combined vaccines.

Viral vector vaccine platforms can be administered as mucosal vaccines through nasal sprays, inducing mucosal immune responses that are long-lasting and cross-reactive. An example is Xia group’s live attenuated influenza vector-based SARS-CoV-2 vaccine (Chen et al., 2022; Zhang et al., 2023). However, the challenge with vector-based vaccines lies in the limited capacity for foreign genes. Currently, most influenza-Covid-19 double-hit vaccines based on this platform are designed based on the SARS-CoV-2 RBD and influenza HA or fusion proteins of RBD with conserved influenza proteins (Ao et al., 2022; Bommireddy et al., 2022; Zhu et al., 2022). To date, no viral vector-based combined vaccine has integrated all four seasonal influenza A subtypes and influenza B lineages along with SARS-CoV-2 or other respiratory viruses. To enhance the efficacy of vector-based vaccines, future research must concentrate on integrating a broader range of antigens.

While pan-respiratory vaccines remain the goal, developing combined vaccines for specific populations is crucial, targeting various respiratory viruses based on age-related immune responses and susceptibilities. For example, vaccines for newborns, children, and the elderly should encompass protection against influenza, SARS-CoV-2, and RSV, reflecting their broader susceptibility, while vaccines for adults may focus on influenza and SARS-CoV-2 due to a lower risk of RSV (McLaughlin et al., 2022b). Additionally, as respiratory viruses like influenza and SARS-CoV-2 evolve over time and vary by location and season, vaccine design must be adaptable to these changing epidemiological patterns. Thus, a vaccine development strategy that considers age-specific needs, and flexibility to include new strains, is essential for effective and broad protection, ultimately improving public health by addressing the dynamic nature of respiratory virus transmission.

## Author contributions

YW: Conceptualization, Writing – original draft, Writing – review & editing. XW: Investigation, Writing – original draft, Writing – review & editing. YL: Writing – review & editing. SL: Investigation, Writing – review & editing. WP: Conceptualization, Writing – review & editing. JD: Writing – review & editing. ZY: Conceptualization, Writing – review & editing.
